# Crystal-Like Atomic Arrangement and Optical Properties of 25La_2_O_3_–75MoO_3_ Binary Glasses Composed of Isolated MoO_4_^2–^

**DOI:** 10.1021/acs.inorgchem.4c00176

**Published:** 2024-03-12

**Authors:** Atsunobu Masuno, Sae Munakata, Yoshihiro Okamoto, Toyonari Yaji, Yoshihisa Kosugi, Yuichi Shimakawa

**Affiliations:** † Graduate School of Engineering, Kyoto University, Nishikyo-ku, Kyoto 615-8520, Japan; ‡ Graduate School of Science and Technology, Hirosaki University, Hirosaki, Aomori 036-8505, Japan; § Institute of Industrial Science, The University of Tokyo, 4-6-1 Komaba, Meguro-ku, Tokyo 153-8505, Japan; ∥ Materials Sciences Research Center, Japan Atomic Energy Agency, 1-1-1 Kouto, Sayo-cho, Sayo-gun, Hyogo 679-5148, Japan; ⊥ Research Organization of Science and Technology, Ritsumeikan University, 1-1-1 Noji-Higashi, Kusatsu, Shiga 525-8577, Japan; # Institute for Chemical Research, Kyoto University, Gokasyo, Uji, Kyoto 611-0011, Japan

## Abstract

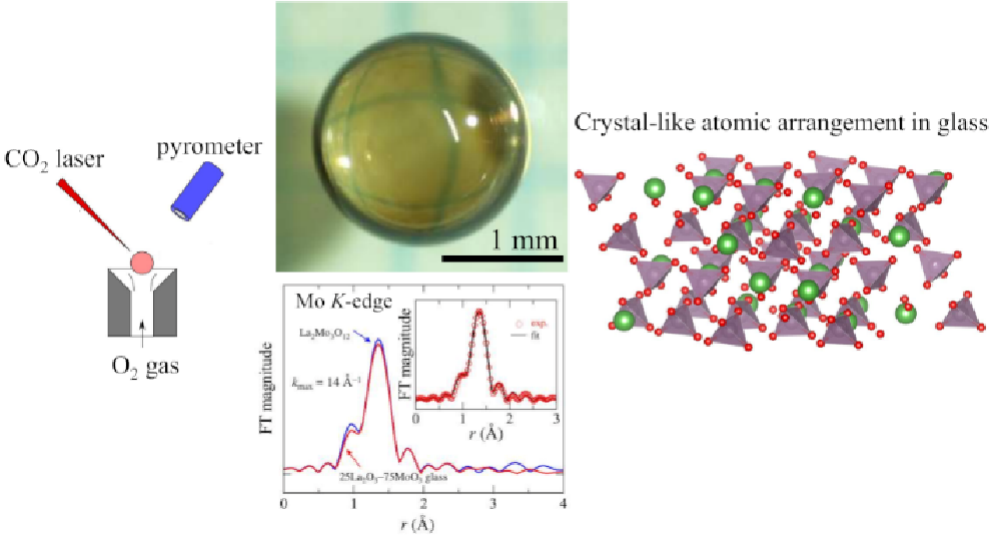

Transparent and brown La_2_O_3_–MoO_3_ binary glasses were prepared in bulk form using a levitation technique. The glass-forming range was limited, with the primary composition being approximately 25 mol % La_2_O_3_. The 25La_2_O_3_–75MoO_3_ glass exhibited a clear crystallization at 546 °C, while determining its glass transition temperature was difficult. Notably, despite its amorphous nature, the glass possessed a density and packing density comparable to those of crystalline La_2_Mo_3_O_12_. X-ray absorption fine structure and Raman scattering analyses revealed that the glass structure closely resembles La_2_Mo_3_O_12_ due to the presence of isolated MoO_4_^2–^ units, whereas disordered atomic arrangement around La atoms was confirmed. The glass demonstrated transparency ranging from 378 to 5500 nm, and the refractive index at 1.0 μm was estimated to be 2.0. The optical bandgap energy was 3.46 eV, which was slightly smaller than that of La_2_Mo_3_O_12_. Additionally, the glass displayed a transparent region ranging from 6.5 to 8.0 μm. This occurrence results from the decreased diversity of MoO_*n*_ units and connectivity of Mo–O–Mo, which resulted in the reduced overlap of multiphonon absorption. This glass formation, with its departure from conventional glass-forming rules, resulted in distinctive glasses with crystal-like atomic arrangements.

## Abstract 2

Transparent and brown La_2_O_3_−MoO_3_ binary glasses were prepared in bulk form using a levitation technique. Mo *L*_3_-edge and *K*-edge X-ray absorption fine structure and Raman scattering spectra revealed that the 25La_2_O_3_−75MoO_3_ glass structure highly resembles crystalline La_2_Mo_3_O_12_, which consists of completely isolated MoO_4_^2−^. The structural similarity with the crystalline phase and the slight disorder caused the additional transparency in the infrared region.

## Introduction

Oxide glasses are constructed on a three-dimensional network of *M*O_*n*_ polyhedra where element *M* and oxygen (O) are bonded covalently. The constraints in glass formation are that the oxygen coordination number, *n*, should be as small as three or four, and that the *M*O_*n*_ polyhedra are connected to each other not by edge- or face-sharing but by corner-sharing. SiO_2_, P_2_O_5_, etc., can form corner-sharing *M*O_4_ tetrahedral networks, and they are thus referred to as network former oxides. Modifier oxides, such as alkali metal oxides, alkaline earth metal oxides, and rare earth oxides, typically break the network and introduce nonbridging oxygens. Al_2_O_3_, TiO_2_, Nb_2_O_5_, and other intermediate oxides act as either a network former or modifier, depending on their composition in a glass.^1,2^ In addition, among the intermediate oxides that cannot vitrify without additives, Al_2_O_3_, Ga_2_O_3_, MoO_3_, and WO_3_ can vitrify relatively easily, even at a binary composition, in combination with a specific modifier or intermediate. They are especially called conditional glass formers.^3^ The glass-forming region of the binary system that includes conditional glass formers is generally narrow. Nevertheless, an additional small amount of network former oxide added to a binary system drastically improves the glass-forming ability.

MoO_3_ is a conditional glass former, and glass formation in some of its binary systems has been reported. However, compared to Al_2_O_3_- and Ga_2_O_3_-based binary glasses, the number of reports is considerably small. Nassau et al. prepared Li_2_O–MoO_3_ binary glasses during a search for glasses with high ionic conductivity in the 1980s.^4^ However, glass formation required the use of the twin-roller quenching method with a very high cooling rate (10^4^–10^6^ K/s). Ag_2_O–MoO_3_ binary glasses with a thickness of approximately 1 mm were obtained by a conventional melt-quench method to investigate their ionic conductivity.^5^ Recent structural analysis combined with X-ray and neutron diffraction revealed that the coordination number of Mo decreased from 5.4 to 4.2 with increasing Ag_2_O content and a variety of MoO*_n_* polyhedra (*n* = 4, 5, and 6) connected to each other to develop the network structure.^6^ Dimitriev et al. have fabricated a variety of binary and ternary glasses that included MoO_3_ as a main component since the 1990s and summarized a large amount of research on nontraditional molybdate glasses.^7−9^ They succeeded in the glass formation of the 10La_2_O_3_–10Nd_2_O_3_–80MoO_3_ and 10*R*_2_O_3_–90MoO_3_ systems (*R* = La or Nd) using slow cooling rates (10^2^ K/s). It was reported that the 10La_2_O_3_–90MoO_3_ glass had the glass transition temperature at around 325 °C and crystallization temperature at 410 °C. From structural analysis using Fourier transform infrared (FT-IR) and X-ray absorption fine structure (XAFS) spectroscopies, it was suggested that the glass network was build up with corner-sharing MoO_6_ or MoO_5_ without short isolated Mo=O bonds.^9^

A levitation technique is an effective method to vitrify a composition without a sufficient network former oxide in bulk form despite its low glass-forming ability. Various unconventional oxide glasses have been fabricated recently using a levitation technique in the last 20 years.^10−13^ New glass compositions and the expansion of the glass-forming region were reported in systems with conditional glass formers as the main component. *R*_2_O_3_–Al_2_O_3_ glasses have very high elastic moduli,^14,15^ and *R*_2_O_3_–Ga_2_O_3_ and *R*_2_O_3_–WO_3_ glasses exhibit good optical properties such as infrared transparency, strong luminescence, and high refractive index with low wavelength dispersion.^16−19^ In this study, La_2_O_3_–MoO_3_ binary glasses were successfully obtained by a levitation technique in the bulk form. Physical properties of the binary glasses, including thermal and optical properties, were measured. Structural analysis using Mo *L*_3_-edge, *K*-edge, and La *L*_3_-edge XAFS and Raman scattering spectroscopies were performed to investigate the atomic arrangement of the glass by comparing it with the crystalline phase structure with the same composition as the glass.

## Experimental Procedure

Glass syntheses of the (100–*x*)La_2_O_3_–*x*MoO_3_ binary system were examined by a levitation technique. High purity La_2_O_3_ and MoO_3_ were stoichiometrically mixed and pressed into pellets. The pellets were sintered at 600 °C for 12 h in air and then were crushed to target pieces for the aerodynamic levitation (ADL) furnace. A piece of the target was placed on the nozzle of the ADL furnace and levitated by an O_2_ gas flow. A CO_2_ laser was used to melt the levitated sample for several seconds. The melt was rapidly cooled to room temperature by turning off the laser power, and then it solidified. The cooling rate was estimated to be approximately 500 °C/sec. Glass formation was confirmed by Cu *K*α X-ray diffraction measurements (XRD: Rigaku, MiniFlex600). Although the size of the fabricated glasses was approximately 2 mm, the thermal, optical, and structural properties could be sufficiently investigated. Crystalline La_2_Mo_3_O_12_ as a reference was prepared by conventional solid-state reaction. High purity La_2_O_3_ and MoO_3_ were stoichiometrically mixed and pressed into a pellet, and it was sintered in air at 800 °C for 24 h several times with intermediate pulverization.

The glass transition temperature (*T*_g_) and crystallization temperature (*T*_*x*_) for the glasses were investigated by using differential scanning calorimetry (DSC: NETZSCH, DSC 404 F1 Pegasus). The temperature was raised from room temperature to 700 °C with a heating rate of 10 °C/min in a N_2_ gas flow. Prior to measurement of the physical properties, the glasses were annealed at 475 °C for 5 min to remove any internal stresses. Furthermore, post annealing of the glass in an oxidized atmosphere was conducted to diminish Mo^5+^ and improve transparency at the 400–600 nm region. The glasses were annealed in an O_2_ gas flow at 400 °C for 24 h. The density of the glasses was measured using a He-gas pycnometer (micromeritics, AccupycII 1340). More than a dozen glass beads were placed in a 0.1 cc sample cell to reduce experimental errors.

Mo *L*-edge XAFS measurements were performed at the soft X-ray beamline, BL-10, in the SR Center of Ritsumeikan University.^20^ The incident X-ray was monochromated with a double crystal using Ge(111) planes. Powdered samples of glasses and reference crystals were put on the carbon seal on the sample holder in a high vacuum chamber. The X-ray absorption spectra were recorded with the fluorescence yield method. Photon energy was calibrated at the energy of a peak (2481.69 eV) of white line of S *K*-edge XANES spectra of K_2_SO_4_ standard samples.

Mo *K*-edge and La *L*_3_-edge XAFS spectra were recorded in transmission mode using two ionization chambers at the BL27B and BL9A beamlines in KEK-PF, respectively.^21−23^ The incident X-ray was monochromated with a double crystal by using Si(111) planes. The samples were ground into powders and mixed with a high purity hexagonal boron nitride powder to form pellet specimens. Energy calibration for Mo *K*-edge spectra was performed by setting the inflection point of the spectra of a metallic Mo foil as *E* = 20003.9 eV and that for the La *L*_3_-edge was done by setting the energy of the pre-edge peak of the Ti *K*-edge of a metallic Ti foil as *E* = 4966.0 eV. XAFS data analysis was conducted using the Athena and Artemis programs in the Demeter software package.^24^ The *k*^3^-weighting EXAFS spectra were Fourier-transformed (FT) to real space. Structural parameters regarding the first peak in the FT magnitude, such as the oxygen coordination number of Mo and La, the cation–oxygen distance, and the Debye–Waller (DW) factor, including both structural and thermal disorder, were evaluated by curve fitting analysis using phases and amplitude functions calculated using the FEFF8 code.

Unpolarized Raman scattering spectra of the glasses and crystalline La_2_Mo_3_O_12_ were obtained in a 180°-scattering geometry by a confocal Raman microscope (JASCO, NRS-4500). The incident source was a 532 nm semiconductor laser.

For transmittance measurements, both sides of the glass samples were optically polished to a thickness of approximately 500 μm. Transmittance spectra were obtained using an ultraviolet–visible (UV–vis) spectrometer (Shimadzu, UV3600 plus) in the range of 250–2000 nm and an FTIR spectrometer (Shimadzu, IRAffinity-1S) in the range of 2000–10000 nm. The diffuse reflectance spectra of powder samples were also obtained using an integrating sphere installed in a UV3600 plus. BaSO_4_ was used as a reference.

## Results and Discussion

**Figure 1:**
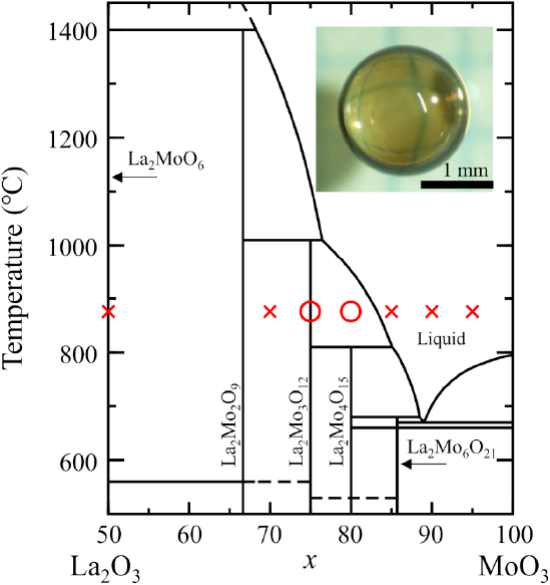
Glass-forming range indicated on the (100–*x*)La_2_O_3_–*x*MoO_3_ phase diagram. Circles and crosses indicate glass formation and crystallization, respectively. The inset shows a picture of the 25La_2_O_3_–MoO_3_ glass annealed in an O_2_ gas flow at 400 °C in 24 h.

The glass synthesis results are shown on the La_2_O_3_–MoO_3_ binary phase diagram^25^ in Figure 1. Bulk glasses were obtained only at *x* = 75 and 80 in the composition of the (100–*x*)La_2_O_3_–*x*MoO_3_. Note that the *x* = 75 glass has the same composition as that of crystalline La_2_Mo_3_O_12_. The glasses were homogeneously transparent and brownish colored, as shown in the inset. The glass-forming region of the La_2_O_3_–MoO_3_ binary system is similar to that of the La_2_O_3_–WO_3_ binary system.^19^ The brown color of the La_2_O_3_–MoO_3_ glasses is a unique characteristic compared to that of colorless La_2_O_3_–WO_3_ glasses. The diameter of the spheric glasses was approximately 2 mm. At *x* = 50, 70, 85, 90, and 95, violent evaporation occurred during melting, and the targets were not vitrified. The glass formation in this study is clearly different from the previous report that showed glass formation with *x* = 90 using the conventional melt-quench method.^7,8,9^ Glass formation at *x* = 90 rarely succeeded in our levitation experiments, but only where the melting time was long and the melting temperature was high. This may be due to composition deviation, resulting in an increase of La_2_O_3_ content because of MoO_3_ volatilization.

**Figure 2:**
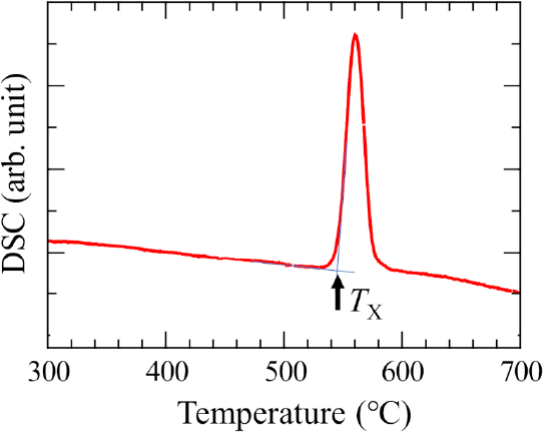
DSC curve of 25La_2_O_3_–75MoO_3_ glass.

Figure 2 shows the DSC curve of the *x* = 75 glass. The crystallization temperature, *T*_*X*_, was found at 546 °C, and the crystallization peak was remarkably sharp. *T*_*X*_ of the *x* = 75 glass was lower than those of 20La_2_O_3_–80WO_3_ glass, which was at 594 °C. Determining the glass transition temperature was challenging due to minimal change in heat flow. Previous reports based on differential thermal analysis (DTA) data of the *x* = 90 glass indicated a crystallization peak temperature at 410 °C, and a subtle change, assumed to be *T*_g_, was observed at approximately 325 °C.^7,9^ However, no significant signal was observed in the temperature range from 300 °C to *T*_*X*_ in this study. Two possible reasons explain why the glass transition temperature was not detectable. One possibility is that *T*_g_ might be so close to *T*_*X*_ that *T*_g_ is obscured by the onset of the crystallization peak. Another possibility is that the difference in specific heat between the glassy state and supercooled liquid might be too small to be detected.

**Figure 3:**
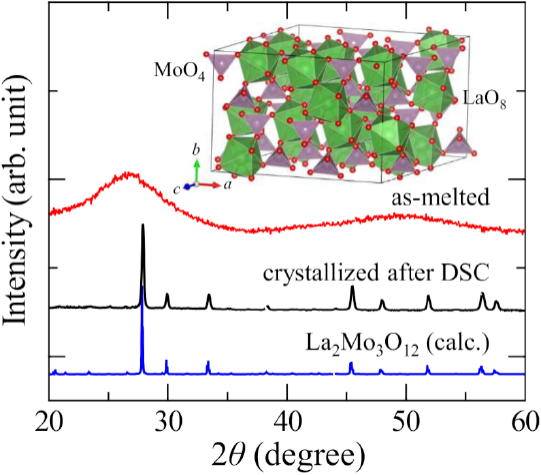
XRD profiles of the as-melted 25La_2_O_3_–75MoO_3_ sample and the crystallized sample after DSC measurement. The calculated profile of La_2_Mo_3_O_12_ is shown at the bottom. The inset shows the crystal structure of La_2_Mo_3_O_12_ drawn using VESTA software.^27^ Purple and green polyhedra in the inset represent MoO_4_ and LaO_8_, respectively.

Figure 3 shows XRD profiles of the as-melted sample of *x* = 75, the crystallized sample after DSC measurement, and a calculated profile using the crystal structure of La_2_Mo_3_O_12_.^26^ The profile of the as-melted sample does not have any peaks, indicative of an amorphous nature. It is clearly shown that the profile of the crystallized sample is almost identical to the calculated profile of La_2_Mo_3_O_12_. This implies that the atomic arrangement of *x* = 75 glass and crystalline La_2_Mo_3_O_12_ may resemble each other. The crystal structure of La_2_Mo_3_O_12_ is shown in the inset of Figure 3. There are five Mo sites and three La sites in the La_2_Mo_3_O_12_ unit cell. The Mo atom forms highly isolated MoO_4_, without any Mo–O–Mo connections, while the La atom is coordinated by eight oxygens. It can be seen that the MoO_4_ tetrahedra connect at all four oxygen with LaO_8_ by corner-sharing. Each of the six oxygens in LaO_8_ is shared with MoO_4_ at the corner, and each of the remaining two oxygens is shared by MoO_4_ and two edge-shared LaO_8_. Alternatively, Mo^6+^ forms isolated MoO_4_^2–^, all of whose oxygen atoms are nonbridging, and La^3+^ coordinates it as a charge compensator.

The density of the *x* = 75 glass was measured to be 4.67 g/cm^3^, indicating a 4.1% deviation from the calculated value of 4.87 g/cm^3^ of La_2_Mo_3_O_12_. The packing density (PD) was calculated using the equation PD = *V*_P_/*V*_m_, where *V*_P_ is the sum of the volume of component ions and *V*_m_ is the molar volume of the glass. Assuming that ions in the glass are spherical, the volume of the *i*th ion is expressed as 4π*r*_*i*_^3^/3, where *r*_*i*_ represents the ionic radius of the *i*th ion. Shannon’s ionic radii were utilized for volume calculations: 1.16 Å for La^3+^(VIII), 0.41 Å for Mo^6+^(IV), and 1.35 Å for O^2–^(II).^28^ The PD of the 25La_2_O_3_–75MoO_3_ glass was determined to be 0.510, closely resembling 0.532 of the La_2_Mo_3_O_12_ crystal. Such similarity in PD between glass and crystalline phase, often observed in glasses prepared by a levitation technique,^29^ suggests that the glass structure is highly packed, akin to the crystalline phase.

X-ray absorption near edge spectroscopy (XANES) spectra mainly originate from a superposition of electronic transitions and provide information on valence and chemical states of the elements to be measured. The relative height and position of the pre-edge peak in XANES spectra are often helpful to estimate the coordination number and distortion of the polyhedron.^30−32^ XANES analyses for Mo were reported for silicate glasses and melts by comparing a variety of reference crystals.^33^ XANES spectral shape and the pre-edge peak of the reference crystals strongly depend on the atomic arrangements and the valence state of Mo. Linear correlation was clearly shown between the average valence state of Mo and the Mo *K*-edge position at a normalized absorbance equal to one. The XANES spectra of silicate glasses also showed this variation; however, the degree of difference is much smaller than the case of crystalline phases. It was concluded that the valence state of Mo was six in silicate glasses prepared in ambient conditions, and the reduction of Mo requires a strong reduced condition. The coordination number of Mo estimated from the pre-edge peaks is almost four; however, there are some glasses with higher coordination numbers, ranging from 4 to 6 even in silicate glasses.^33^

**Figure 4:**
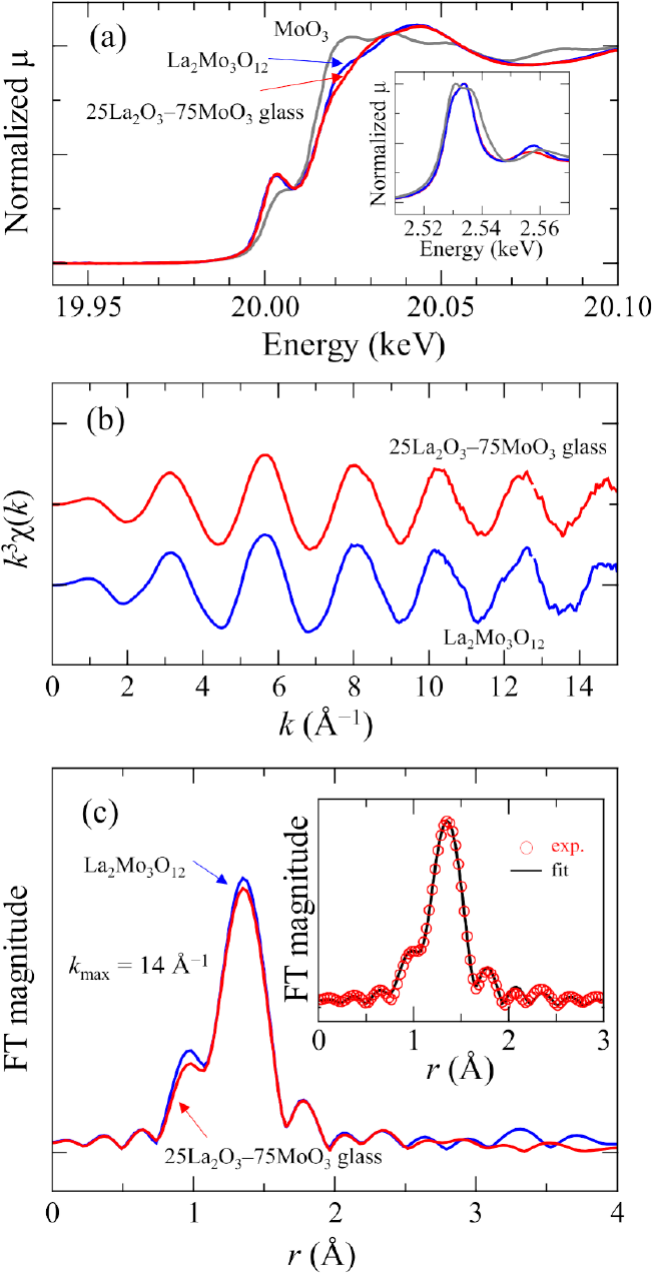
Mo *K*-edge XAFS spectra of 25La_2_O_3_–75MoO_3_ glass and crystalline La_2_Mo_3_O_12_. (a) XANES spectra, (b) *k*^3^-weighted EXAFS spectra, and (c) the FT magnitude of EXAFS oscillation. Inset of (a) shows Mo *L*_3_-edge XANES spectra. Inset of (c) shows the fitting result of the glass.

Mo *K*-edge XAFS spectra of the La_2_O_3_–MoO_3_ binary system were clearly different from silicate glasses, and they provide clear evidence of the similarity of local structure around Mo atoms between glassy and crystalline phases. Figure 4a shows Mo *K*-edge XANES spectra of the 25La_2_O_3_–75MoO_3_ glass and La_2_Mo_3_O_12_ with reference MoO_3_. Mo in MoO_3_ has a valence state of 6+ and is coordinated by six oxygen atoms. MoO_6_ polyhedra are connected to each other by sharing corners and edges. The spectrum of the glass is clearly different from that of MoO_3_, especially at the pre-edge region; however, it is almost the same as that of La_2_Mo_3_O_12_. Compared to MoO_3_, the pre-edge peak of the glass is higher, and the position shifted to the lower energy side, indicating that Mo forms MoO_4_ tetrahedra as well as La_2_Mo_3_O_12_. From the absorption edge energy, Mo in the glass is estimated to be 6+, as well as that in the two reference crystals.

Mo *L*_3_-edge XANES spectrum, in which the peaks are mainly ascribed to the transition from 2*p* to 4*d* states, is helpful to predict the oxygen coordination number of Mo as well as *K*-edge XANES spectrum.^34^ Mo *L*_3_-edge XANES spectra of the glass and reference crystals are shown in the inset of Figure 4a. Two peaks were clearly observed in the spectra of MoO_3_. The peak at the higher energy side was less intense than that on the lower energy side, implying that they are associated with the *e*_g_ and *t*_2g_ empty states of octahedral MoO_6_. The spectra of the glass and crystalline La_2_Mo_3_O_12_ were almost the same shape, which is evidence of the local structural similarity between them. The main peak consists of two peaks with an energy difference smaller than that of the MoO_3_ spectrum. The intensity ratio of the peak at the higher energy side and lower side was the opposite of that of MoO_3_. These results suggest that the two peaks are attributed to the electron transition in the tetrahedral crystal field splitting.

Figure 4b shows the *k*^3^-weighted EXAFS spectra, *k*^3^χ(*k*) of the 25La_2_O_3_–75MoO_3_ glass and crystalline La_2_Mo_3_O_12_. The oscillation was observed clearly in a high *k* range. The glass and crystal are almost the same in shape and intensity. This means the homogeneity and uniformity of the local structure around Mo in the glass. Figure 4c shows the FT amplitude of the *k*^3^-weighted EXAFS spectra with *k*_max_ = 14.0 Å^–1^. They are almost the same; however, a relatively smaller peak height was observed for the glass, indicating a slightly smaller coordination number or disorder in atomic arrangement around Mo. Furthermore, there is no correlation at 3.8–4.0 Å for the second nearest shell in the glass, while a small but clear correlation is observed in the crystal, showing disorder in the glass. A correlation smaller than expected from the FEFF calculation is often observed in molybdate crystalline phases, such as Na_2_MoO_4_ and Ag_2_MoO_4_.^33,35^

In order to more quantitatively investigate the local structure around the Mo, fitting of the *K*-edge EXAFS spectra was performed. Prior to the fitting of EXAFS spectra for the glass, it is necessary to determine the inelastic multielectronic losses, *S*_0_^2^, of the EXAFS formula in this experiment from the fitting result of a reference crystal. The fitting for La_2_Mo_3_O_12_ was performed in the *r* range from 1 to 2 Å with a fixed value of 4.0 for the coordination number, *N*. The value of *S*_0_^2^ was 0.885. The Mo–O distance, *R*, and DW factor, σ^2^, are 1.778 ± 0.002 and 0.0022 ± 0.0002 Å^2^, respectively, as shown in Table 1. The fitting of the glass was performed by using the *S*_0_^2^ value. The coordination numbers of Mo, *R*, and σ^2^ were 3.9 ± 0.1, 1.778 ± 0.002 Å, and 0.0022 ± 0.0002 Å^2^, which are almost the same as that of La_2_Mo_3_O_12_. The coordination number of the glass certainly smaller than that of the crystal but they are almost the same within an error. It should be noted that there is almost no difference in the structural parameters *N*, *R*, and σ^2^ between the glass and crystal, which strongly indicates the similarity in the local structure of the Mo atom in the first coordination shell. Therefore, it is highly estimated that Mo in the glass forms regular tetrahedra with four nonbridging oxygens as in the case of La_2_Mo_3_O_12_.

**Table 1:** Parameters Used to Fit the Mo *K*-edge EXAFS Spectra of 25La_2_O_3_–75MoO_3_ Glass and Crystalline La_2_Mo_3_O_12_

	*N*	*R* (Å)	σ^2^	Δ*E* (eV)
25La_2_O_3_–MoO_3_ glass	3.9(1)	1.778(2)	0.0022(2)	2.1(7)
crystalline La_2_Mo_3_O_12_	4.0^a^	1.778(2)	0.0022(2)	2.4(7)

a Fixed value, S_0_^2^ = 0.885.

**Figure 5:**
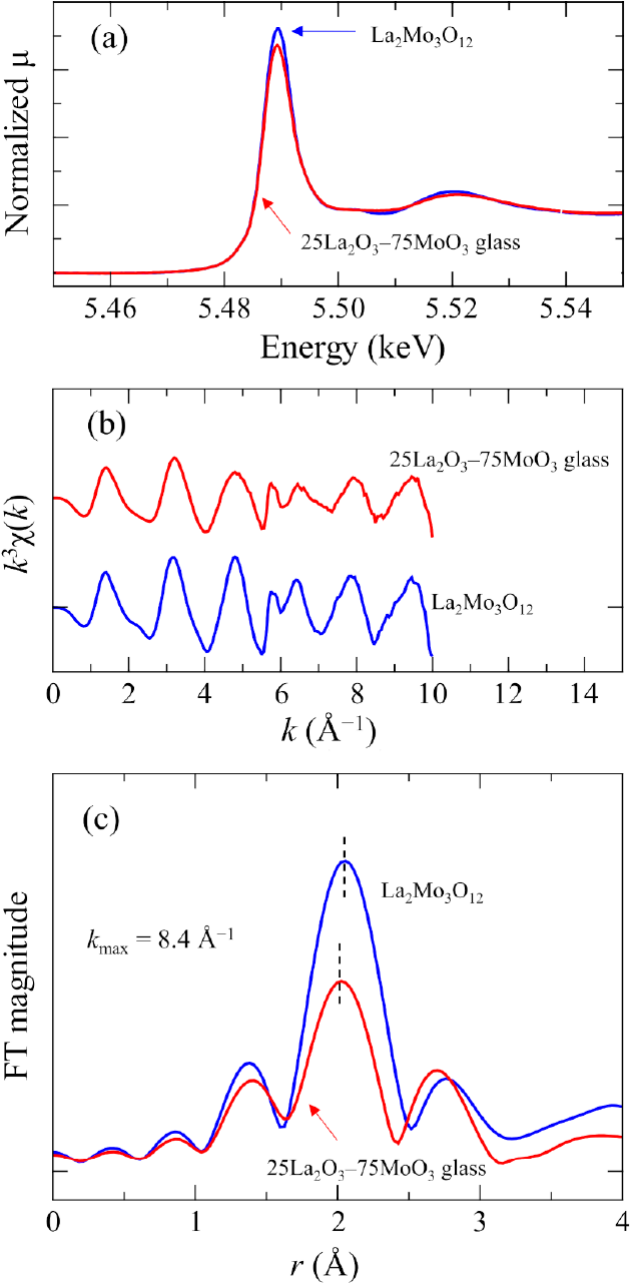
La *L*_3_-edge XAFS spectra of 25La_2_O_3_–75MoO_3_ glass and crystalline La_2_Mo_3_O_12_. (a) XANES spectra, (b) *k*^3^-weighted EXAFS spectra, and (c) the FT magnitude of the EXAFS oscillation.

Figure 5 shows La *L*_3_-edge XAFS spectra of 25La_2_O_3_–75MoO_3_ glass and crystalline La_2_Mo_3_O_12_. In contrast to Mo *K*-edge XAFS, there is a clear difference between the glass and the crystal. The peak height of the white line in the glass XANES spectrum is clearly smaller than that of the crystal, and the oscillation above 5.50 eV for the glass is not obvious compared to the crystal (Figure 5a). This is indicative of the disordered structure of the glass. Figure 5b shows EXAFS spectra of the glass and the crystal. The intensity of the glass is much smaller than the crystal; however, the oscillation periods are similar to each other, indicative of local environment similarity around the La atoms. Because the La *L*_2_-edge absorption is nearly located, there is a limited *k* range available for FT of the EXAFS spectra. Figure 5c shows the FT magnitudes of the glass and the crystal. Because of the limited range of *k* (3 Å^–1^ ≤ *k* ≤ 8.4 Å^–1^), it is difficult to estimate structural parameters by fitting. From the simple fitting of crystalline spectra with a fixed coordination number value of eight, the inelastic multielectronic loss, *S*_0_^2^, is estimated to be 0.95, and the La–O distance *R* and DW factor σ^2^ are 2.48 ± 0.02 Å and 0.007 ± 0.001 Å^2^, respectively. By using the *S*_0_^2^ value, the coordination number of La, *R*, and σ^2^ for the glass were 6.4 ± 0.1, 2.46 ± 0.01 Å, and 0.009 ± 0.002 Å^2^, respectively. The La–O bond length of the glass is slightly shorter than that of the crystal. The apparent small coordination number of glass might be due to disorder in the atomic arrangement and does not indicate that the number of oxygen atoms around La in the glass is smaller than that of the crystal. The disorder sometimes diminishes the longer side peak by averaging the distribution in amorphous materials. Accordingly, XAFS analysis using Mo *K*-edge and La *L*_3_-edge absorptions indicates structural similarity around Mo and La between the glass and crystal, although disorder is obvious, especially around La in the glass.

**Figure 6:**
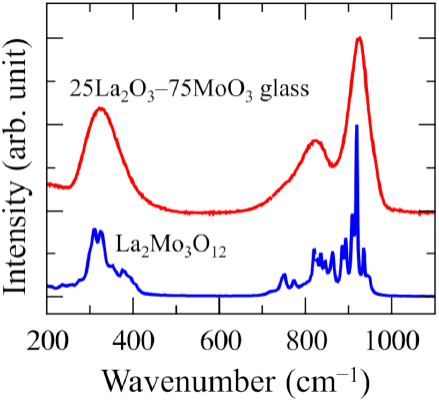
Raman scattering spectra of 25La_2_O_3_–75MoO_3_ glass and crystalline La_2_Mo_3_O_12_.

Further evidence of the structural similarity between the glass and the crystal is clearly shown in the vibration spectra. Figure 6 shows Raman scattering spectra of the glass and the crystal. It should be noted that the spectrum of glass clearly resembles the crystalline spectrum in peak position and peak height. It seems that broadening the spectrum of the crystal matches well with that of the glass. The agreement of the glass and the crystal strongly suggests that the local environment around the Mo atoms, such as the bonding distance to oxygen, the oxygen coordination number, and the oxygen–cation–oxygen bond angles, is almost the same. Detailed analyses of Raman bands of crystals including Mo have been reported in the literature.^36−38^ The bands at 880–970 cm^–1^ and 750–880 cm^–1^ are attributed to the Mo–O symmetric stretching mode and antisymmetric stretching modes in MoO_4_ tetrahedra, respectively. The band at 320 cm^–1^ is attributed to the O–Mo–O bending mode in MoO_4_ tetrahedra.

**Figure 7:**
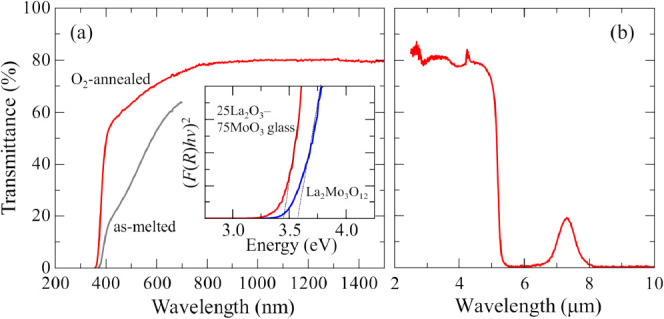
(A) UV–vis transmittance spectra of 25La_2_O_3_–75MoO_3_ glasses. Red line represents the glass annealed in O_2_ gas flow, while gray line represents the as-melted glass. (Inset) Tauc plot using the Kubelka–Munk function, *F*(*R*), obtained from diffuse scattering spectra of 25La_2_O_3_–75MoO_3_ glass and crystalline La_2_Mo_3_O_12_. The factor *m* is 1/2. (b) IR transmittance spectra of a 25La_2_O_3_–75MoO_3_ glass.

Finally, the optical properties of the glass are shown in Figure 7. Figure 7a shows the transmittance spectra of the *x* = 75 glasses in the UV–vis region. Compared with the spectrum of the as-melted glass, the transparency of the glass annealed in the O_2_ gas flow was improved in the visible range. The UV absorption edge is approximately 378 nm, from which the optical bandgap energy is estimated to be 3.40 eV. The small absorption at 400–700 nm is obvious, which might be due to the *d*–*d* transition in Mo^5+^. The amount of Mo^5+^ should be very small because it was difficult to find evidence of a lower valence state of Mo in the Mo *K*-edge XANES spectra. The maximum transmittance was 80% at 1000 nm, which can be attributed to reflection on both sides of the surface during the measurement. Assuming that there is only surface reflection and no light scattering inside the glass, the refractive index, *n*, of the glass is estimated to be 2.0 using the equation *T*_max_ = 1 – [2*R*′/(1 + *R*′)], where *T*_max_ is the maximum transmittance and *R*′= [(*n* – 1)/(*n* + 1)]^2^. The estimated refractive index at 1000 nm is slightly larger than that of 20La_2_O_3_–80WO_3_ glass.^19^ Since the optical bandgap energy of the *x* = 75 glass is not so large, the wavelength dispersion of the refractive index will be large, which is not suitable for lenses in the visible region. Nevertheless, the La_2_O_3_–MoO_3_ glasses are possible candidates for optical applications using high refractive index in the near-infrared region.

The optical absorption edge of the glass can be compared to that of the powdered crystalline phase using diffuse reflectance spectroscopy. The Kubelka–Munk function, *F*(*R*), is obtained from the reflectance spectra. The Tauc method equation is transformed to eq 1 by substituting *F*(*R*) for the absorption coefficient, α:
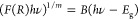
1where *h* is the Planck constant, *ν* is the photon frequency, *E*_g_ is the optical band gap energy, and *B* is a constant. The factor *m* depends on the nature of the electron transition and is equal to 1/2 or 2 for direct or indirect transition band gaps, respectively. The inset of Figure 7a shows the Tauc plot with *m* = 1/2 by using the Kubelka–Munk function. The Tauc plot curve of the glass clearly shifts to the lower energy side compared to that of the crystalline sample. The *x*-axis intersection point of the linear fit of the Tauc plot gives an optical band gap energy. The estimated *E*_g_ of the glass is 3.46 eV, which is in good agreement with the value obtained from transmittance spectra. The *E*_g_ of the crystalline sample is 3.60 eV.^39^ The bandgap of the glass is slightly smaller than that of the crystal, which is reasonable considering the disorder in atomic arrangements characteristic to amorphous materials.

Figure 7b shows the infrared transmittance spectra of the glass. The glass was transparent to 5.5 μm, and the absorption at 2.9 μm is attributed to the presence of the O–H vibration. Compared with conventional oxide glasses, the O–H absorption of the glass is considerably small, indicating a scarcity of OH groups. Furthermore, there is the additional transmittance window in the range 6.5–8.0 μm. A transparent region at the wavelength side longer than the infrared absorption edge was also found in *R*-rich *R*_2_O_3_–B_2_O_3_ binary glasses (*R* is a rare earth element or Y).^40−42^ The additional transmittance in the *R*-rich borate glasses was explained by considering the local structure around the B atoms. B atoms in the glass form isolated BO_3_ units without any three-dimensional network structure. The simple environment around the B atoms resulted in the suppression of the overlap of the absorption bands of the B–O multiple vibrations and their overtones observed in conventional borate glasses. As a result, the absorption bands discretize, and then an additional transmittance window emerges. The local structure around the Mo atom in the 25La_2_O_3_–75MoO_3_ glass is confirmed to be the isolated MoO_4_^2–^ from XAFS and Raman scattering spectra, which is seen in crystalline La_2_Mo_3_O_12_. Therefore, the additional transmittance window at 6.5–8.0 μm can be explained by suppression of the overlap of the absorption bands of Mo–O multiple vibrations and their overtones because of the simple environment around Mo as in the case of crystalline La_2_Mo_3_O_12_.

Crystallization of the glass, XAFS and Raman scattering spectra, and the additional infrared transmittance as mentioned above strongly indicate that the atomic arrangement of the 25La_2_O_3_–75MoO_3_ glass is almost the same as that of the crystalline La_2_Mo_3_O_12_, even though structural disorder in the glass was confirmed in the La *L*_3_-edge XAFS and the absorption edge in the UV–vis region. MoO_3_ is classified as a conditional network former, and it has been reported that MoO*_n_* (*n* = 4, 5, or 6) formed a network in MoO_3_-based glasses.^5,6^ Contrary to previously reported glasses, there is no network in the La_2_O_3_–MoO_3_ glasses and Mo forms completely isolated MoO_4_^2–^. Furthermore, the La_2_O_3_–MoO_3_ glasses are highly packed and comparable to those of the crystalline phase. The highly dense packed structure and the structural similarity of the crystal are unique and distictive in glass science; however, similar results have been reported recently in glasses prepared by a levitation technique.^12,40^ As suggested in the previous study, the structural similarity of the glass and the crystalline phases might enhance the glass-forming ability of the densely packed glass system. Assuming that *R*^3+^ and O^2–^ are arranged in the nearly closest packed structure and small cations, such as Mo^6+^ ions, are inserted into interstitial sites (tetrahedral sites in the case of La_2_O_3_–MoO_3_ binary system) in the glass, the amorphous nature is produced by the slight displacement from the atomic arrangement of the crystalline phase. The small displacement enables a fall in metabasins in the free energy landscape^43,44^ and thus loses long-range order in the case of densely packed glass systems.^45^ In this case, glass formation becomes possible even when the three-dimensional regular tetrahedral networks are not developed.

## Summary

Bulk glass formation of the La_2_O_3_–MoO_3_ binary system was achieved using the levitation technique. The glass-forming region was narrow at the vicinity of 25-mol % La_2_O_3_. The fabricated glasses were brownish but transparent in the visible region. The physical and structural properties of the 25La_2_O_3_–75MoO_3_ glass were investigated because competing crystalline phase of La_2_Mo_3_O_12_ exists at the same composition. The glass transition temperature was not detected due to minimal change in heat flow, while the crystallization temperature was 546 °C. After the sharp crystallization peak, a single phase of La_2_Mo_3_O_12_ crystallized. Mo *L*_3_-edge and *K*-edge XAFS and Raman scattering spectra revealed structural similarity between the glass and the crystal. The glass is formed by isolated MoO_4_^2–^ tetrahedra without any network formation. La *L*_3_-edge XAFS and optical bandgap in the UV–vis range indicate the disorder in the atomic arrangement of the glass. An additional infrared transparent window at 6.5–8.0 μm was explained by the simple environment of the local structure around Mo, which has been commonly observed in highly packed glasses prepared by the levitation technique. The estimated refractive index of 2.0 at 1.0 um and broad infrared transparency open up the possibility of optical applications in the infrared range. Furthermore, it was suggested that the glass formation of the La_2_O_3_–MoO_3_ system was caused by slight movement from the ordered La_2_Mo_3_O_12_ structure and, thus, it does not require a three-dimensional tetrahedral network.
